# Reducing the Energy Consumption of sEMG-Based Gesture Recognition at the Edge Using Transformers and Dynamic Inference

**DOI:** 10.3390/s23042065

**Published:** 2023-02-12

**Authors:** Chen Xie, Alessio Burrello, Francesco Daghero, Luca Benini, Andrea Calimera, Enrico Macii, Massimo Poncino, Daniele Jahier Pagliari

**Affiliations:** 1 Department of Control and Computer Engineering, Politecnico di Torino, 10129 Turin, Italy; 2 Interuniversity Department of Regional and Urban Studies and Planning, Politecnico di Torino, 10129 Turin, Italy; 3 Department of Electrical, Electronic and Information Engineering, University of Bologna, 40136 Bologna, Italy; 4 Department of Information Technology and Electrical Engineering, ETH Zurich, 8092 Zurich, Switzerland

**Keywords:** transformers, sEMG, gesture recognition, deep learning, embedded systems

## Abstract

Hand gesture recognition applications based on surface electromiographic (sEMG) signals can benefit from on-device execution to achieve faster and more predictable response times and higher energy efficiency. However, deploying state-of-the-art deep learning (DL) models for this task on memory-constrained and battery-operated edge devices, such as wearables, requires a careful optimization process, both at design time, with an appropriate tuning of the DL models’ architectures, and at execution time, where the execution of large and computationally complex models should be avoided unless strictly needed. In this work, we pursue both optimization targets, proposing a novel gesture recognition system that improves upon the state-of-the-art models both in terms of accuracy and efficiency. At the level of DL model architecture, we apply for the first time tiny transformer models (which we call *bioformers*) to sEMG-based gesture recognition. Through an extensive architecture exploration, we show that our most accurate bioformer achieves a higher classification accuracy on the popular Non-Invasive Adaptive hand Prosthetics Database 6 (Ninapro DB6) dataset compared to the state-of-the-art convolutional neural network (CNN) TEMPONet (+3.1%). When deployed on the RISC-V-based low-power system-on-chip (SoC) GAP8, bioformers that outperform TEMPONet in accuracy consume 7.8×–44.5× less energy per inference. At runtime, we propose a three-level dynamic inference approach that combines a shallow classifier, i.e., a random forest (RF) implementing a simple “rest detector” with two bioformers of different accuracy and complexity, which are sequentially applied to each new input, stopping the classification early for “easy” data. With this mechanism, we obtain a flexible inference system, capable of working in many different operating points in terms of accuracy and average energy consumption. On GAP8, we obtain a further 1.03×–1.35× energy reduction compared to static bioformers at iso-accuracy.

## 1. Introduction

Nowadays, edge computing on energy-efficient devices is a major trend in the personalized healthcare field, with benefits such as low cost, portability, and real-time health data analysis. Many applications, including heart rate (HR) monitoring, blood oxygen saturation measurement, and early warning of fall risk, have been moved to wearable devices and supported by direct execution at the edge. With respect to the traditional approaches based on cloud computing, the on-device paradigm reduces the total energy consumption by eliminating raw data transmission. Moreover, by getting rid of the dependency on a good-quality connection at all times, it ensures fast and predictable response times, making more and more novel healthcare and wellness applications achievable [1,2].

On the same trend, human–machine interfaces (HMIs) based on hand gesture recognition have shown great potential, enabling various novel types of interactions with robots and computers, especially useful for physically impaired subjects [3] and hearing impaired ones [4]. A popular approach is vision-based gesture recognition, which classifies videos of the hand gesture captured by optical cameras [5,6]. However, this approach relies on the quality of visual frames, and classification results can be negatively affected by environmental factors, such as camera position, obstructions, light conditions, and so on. On the other hand, gesture recognition based on sEMG signals, which can directly reflect behavioral intention from muscle contractions at different arm positions, is receiving increasing interest from the scientific community. Compared to the vision-based alternative, this solution is more invasive, requiring the user to wear a potentially uncomfortable set of electrodes on their arm (e.g., [7,8] shows an example of the physical interface required). However, it benefits from wearability and smaller raw input data bandwidth and is less affected by environmental factors. Moreover, sEMG-based solutions are not restricted to specific camera-equipped environments and also enable the recognition of gesture intentions of amputees.

Most current sEMG-based gesture recognition systems utilize classic machine learning (ML) algorithms coupled with carefully crafted feature extraction [8,9,10,11]. Selecting appropriate features to achieve the best possible classification performance in all conditions is known to be a hard problem. Therefore, in recent years, DL algorithms, which learn feature representations during training, have drawn increasing attention and have become the state-of-the-art approach for this task [4,12,13,14].

The implementation of a DL-based system at the edge comes with significant challenges. Most advanced DL algorithms were initially designed for the cloud, and are too energy-hungry and complex to deploy on memory- and energy-constrained edge devices. In fact, the latter are typically battery-operated, they have power envelopes in the order of tens of mW, and are equipped with less than 1MB of working memory (SRAM) [15]. Thus, they cannot support the large memory footprint and huge amount of operations required by cloud models [12,14,16]. To fill this gap, lightweight yet accurate DL models specifically tailored for edge devices have been proposed. Optimization techniques at different levels can be adopted to generate such models, ranging from modifications of the architecture through architectural search and pruning [17] to reductions in the bit-widths used to represent weights and intermediate activations with quantization [18]. Thanks to these techniques, colossal DL models can be slimmed down to fit edge devices, while retaining superior performance with respect to classic ML algorithms based on handcrafted features.

However, since innovation in DL architectures is booming in recent years, there is typically a temporal lag between the initial design of a new cloud model and its porting at the edge. One representative of this trend is the transformer, a recent DL architecture initially designed to solve sequence-to-sequence problems [19], which nowadays achieves state-of-the-art performance in many applications in the fields of natural language processing (NLP) and computer vision (CV), through popular implementations such as BERT [20], GPT-3 [21], and vision transformers (ViT) [22]. All these popular transformers are cloud-based, and include millions or billions of parameters, which makes them impossible to deploy at the edge. On the other hand, most research on edge deployment and optimization still concentrates on “older” models, such as CNNs, and relatively few edge-oriented transformers have been proposed.

In this work, which extends [23], we demonstrate that efficient transformers specifically tailored for edge devices are able to achieve very high energy efficiency, while maintaining state-of-the-art performance. We focus in particular on using these networks for the sEMG-based gesture recognition task. In addition, we also leverage *dynamic inference* techniques [2] to further increase the flexibility and efficiency of our proposed gesture recognition system. Rather than always processing all inputs with the *same* transformer model, we combine two different networks, and a third, non-deep classifier, to form a three-stage system capable of significantly reducing the average energy consumption per classification, at the cost of a negligible drop in accuracy. To our knowledge, this is the first application of dynamic inference both to the sEMG-based gesture recognition task, and to tiny transformers deployed at the edge. The main novel contributions of our work are the following:

- We introduce *bioformers*, a set of efficient transformer architectures, which can achieve state-of-the-art accuracy on a popular sEMG-based gesture recognition dataset [7]. We perform an extensive network architecture exploration, varying several key hyper-parameters of our bioformers, such as the number of initial convolutional layers, the dimension of the input signal patches passed to the attention layers, the number of attention blocks, the dimension and number of attention heads, etc. We obtain several Pareto-optimal configurations, achieving different trade-offs in terms of accuracy versus computational complexity. Specifically, the accuracy ranges from 62.4% to 69.8%, outperforming the 66.7% obtained by a state-of-the-art CNN [24] on the same data and with the same training protocol.
- We propose a novel *multi-stage dynamic inference* scheme to achieve further energy reductions and to improve the flexibility of our gesture recognition system. Specifically, in a first stage, a lightweight RF separates inputs relative to a gesture from those corresponding to a rest condition (no gesture). Only when a gesture is predicted, a small bioformer is invoked to classify it. Then, based on a measure of the classification’s confidence, the process is either stopped at this second stage, or continued, invoking an additional, larger bioformer.
- When deployed on the GAP8 ultra-low-power SoC [15], bioformers achieve an execution time of 0.36–2.80 ms per classification, while consuming 19–143 μJ, and requiring at most 104 kB of memory. Bioformer configurations that achieve a higher quantized accuracy compared to the CNN of [24] consume 7.8×–44.5× less energy per inference. Moreover, thanks to the proposed dynamic inference scheme, we obtain a system that can be configured at runtime to work in 10 s of different operating points, spanning an ample accuracy range (60.9-69.8%). On GAP8, dynamic solutions further reduce the average energy consumption per classification by 1.03×–1.35× at iso-accuracy compared to static bioformers.

## 2. Background

### 2.1. Attention and Transformers

In [19], the authors introduce the transformer architecture, which leverages the *attention* mechanism to enhance the performance of a DL model on sequence processing tasks. Attention is based on the intuition that, in an input sequence, not all the elements are equally important for the target task. Thus, a subset of the inputs should have higher impact on the final output (the network should pay “close attention” to them), while other parts could almost be disregarded. The layers that model this mechanism in neural networks compute a set of input-dependent weights (so-called *attention weights*), whose values correspond to the relative importance of different input sequence portions.

More precisely, the authors of [19] use a variant called multi-head self-attention (MHSA). The name self-attention refers to the fact that importance weights are computed as a function of the same sequence to which they are applied, as opposed to cross-attention, which considers two different sequences. Furthermore, MHSA splits the attention function in *H* separate representations, the so-called *heads*, with the goal of leveraging the information from different representation subspaces. In this work, we leverage MHSA blocks to implement our efficient DL models for sEMG-based gesture recognition.

As main reference implementation, we take the one of the vision transformers (ViTs) [22], which extended attention-based DL models to work with patches of image pixels instead of natural language tokens. In our case, we follow a similar approach, but feed the MHSA with patches extracted from the sEMG signal time-series.

### 2.2. Surface Electromyographic Signal

Elecromiography (EMG) signals stem from the electrical activity generated during the contraction of a muscle, and are typically collected by placing conductive plates (electrodes) on the subject’s skin surface (surface EMG or sEMG). The resulting electrical signals range from 10 μV to 1mV, with a bandwidth of ≈2 kHz for standard applications, or up to ≈10 kHz if the EMG signal is collected for motor unit action potential analysis.

Recognition applications based on these signals must tackle several major challenges, such as the high variability and often low signal quality due to the data acquisition through the less-than-optimal skin–electrode interface. Moreover, as explained by the authors of [24], floating ground noise and motion artifacts, together with possible electrode re-positioning and user adaptation, may cause further signal degradation.

Accordingly, current state-of-the-art gesture recognition systems that rely on sEMG signals use subject-specific ML or DL models, trained on data from the same person on which they are evaluated or pre-trained on a wider set of patients and then fine-tuned on subject-specific data. In order to account for the time drift of sEMG signals caused by the aforementioned non-idealities, the generalization capabilities of these systems should then be assessed in terms of how well they perform on data collected in *different recording sessions* with respect to those used for training, as detailed in Section 3.

## 3. Related Work

In recent years, many approaches for hand gesture recognition using sEMG signals have been developed for both academic and commercial purposes. These approaches typically consist of three parts: (i) an analog front end for bio-potential acquisition, (ii) a data preprocessing and feature extraction/selection step, and (iii) a final classification back end. They often use classic ML algorithms, such as support vector machines (SVMs), RFs, linear discriminant analysis (LDA), or artificial neural networks (ANNs) [8,9,10,11,13], or more recently, DL ones [4,23,24,25,26,27]. For example, authors in [28,29] have achieved over 90% accuracy in hand gesture classification using ANNs with five time-domain features (mean absolute value, mean absolute value slope, number of slope sign changes, number of zero crossings, and waveform length). Another study [30] achieved 97.1% classification accuracy on the recognition of three grasps using root mean square values as features for an SVM. On a more general scenario (three datasets with tens of gestures), remarkable results were obtained by Atzori et al. [8] on the NinaPro DB1, DB2, and DB3 datasets, employing both time- and frequency-domain-extracted features. However, these approaches are limited to a single-session setup, which does not address the issue of inter-session accuracy drop when classifying gestures from a never-seen session.

The main challenge in sEMG-based gesture recognition has therefore shifted from achieving high classification accuracy to managing the variability of the signal, which is affected by various factors, such as anatomical variability, posture, fatigue, perspiration, changes in the skin-to-electrode interface, user adaptation, and electrode repositioning over multiple days of use [7,9,23,24,31]. These factors significantly hinder generalization, limiting long-term use and the development of robust real-time recognition systems. For instance, [7,32] collected sEMG data from multiple subjects over multiple days and found that the inter-session accuracy drop for conventional ML algorithms was up to 30% after training on a single session. To overcome these challenges, a new state-of-the-art approach is to use multi-session training, which aims to make recognition more robust against temporal variability. This approach has been made possible by the availability of multi-session sEMG datasets such as NinaPro DB6 (10 sessions, 8 classes) [7], which is the dataset used in this work, and is described in detail in Section 4.1. In Table 1, we report the most relevant works from the literature that used Ninapro DB6 as a benchmark. Under the accuracy column, we report as *inter-session* accuracy the accuracy achieved on unseen, consequent in time sessions; as *intra-session*, the accuracy on the same data sessions used for training, but following a temporal data split (the initial part of a session is used for training, and the final part for validation); and as *random*, the accuracy on a random data split at the level of individual samples.

In Palermo et al. [7], which first introduced the new dataset, the authors reached an inter-session accuracy of 25.4% by feeding the waveform length feature to an RF. Successively, Cene et al. [33] employed extreme learning machines (ELMs) to raise the inter-session accuracy to 41.8%. In 2019, Wei et al. [25] first applied DL to this dataset, reaching 64.1% inter-session accuracy by feeding raw data to a multi-view CNN. Similarly, Zanghieri et al. [24] employed a temporal convolutional network (TCN) that reached a new state-of-the-art accuracy of 65.2% (61.0% when quantized to 8-bit integers). In 2021, Zou et al. [26] and Han et al. [27], used the NinaPro DB6 as a benchmark, but they picked random data or random sessions to create training, validation, and test datasets, not following any temporal order and therefore reaching very high accuracy; however, this is not representative of a real-world setup. It is worth noticing that the reason why the accuracy reached on the NinaPro DB6 is much lower than the one reached on other datasets with a similar number of classes and sensors is that the hand movements of NinaPro DB6 are all grasps, thus much less diverse and discernable than the gestures in ordinary datasets.

Few of the aforementioned works focus on achieving real-time classification, or on embedding these algorithms on low-power edge devices, such as wearables. In [24], the authors show that their algorithm fits the real-time classification constraint of 15 ms, needed to have fluid movement, for instance, in an artificial-intelligence-powered prosthesis, with an energy consumption per classification of 9.8 mJ.

Tiny transformers were first applied to this task in our previous work [23], where we demonstrated that they can reach a state-of-the-art accuracy of 65.73% (64.7% with int8 data representation) with an energy consumption of just 0.143 mJ per classification, dramatically increasing the battery lifetime of the edge device on which the network is deployed compared to the CNN of [24]. In this work, we further extend this approach, by improving the accuracy of ∼4%, while keeping a constant energy consumption. Furthermore, we also show how our tiny transformers can be combined with a dynamic inference system to obtain additional energy savings and flexibility, at zero (or very limited) cost in accuracy.

## 4. Material and Methods

**Figure 1:**
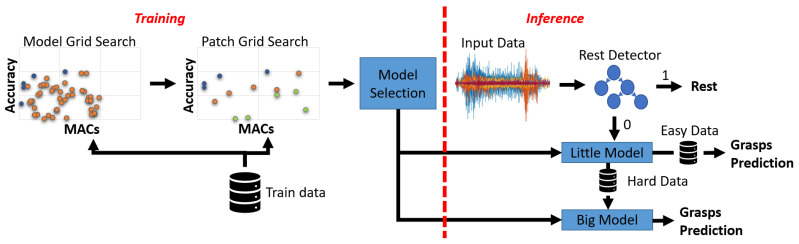
High-level view of the proposed methodology.

The goal of this paper is to propose energy-efficient sEMG gesture recognition systems that can run at the edge, based on transformer neural networks. The overall methodology that we follow to derive such systems is depicted in Figure 1, and consists of two main parts.

The first part (left side of the Figure) is performed at design time, and consists in exploring the space of possible transformer architecture hyper-parameters in order to identify a Pareto-optimal set of solutions in terms of accuracy versus model size, and accuracy versus number of multiply-and-accumulate (MAC) operations, where the two non-functional metrics are proxies for memory occupation and latency/energy consumption, respectively. We call the tiny attention-based models derived from this search phase “Bioformers”, since they are one of the first applications of transformers to biosignal processing. After detailing the sEMG dataset (Ninapro DB6) that we use for benchmarking our work in Section 4.1, we describe the search space explored in this first phase in Section 4.2, while Section 4.3 provides the details of the procedure used to train bioformers.

The second part of the methodology (right side of Figure 1) deals with runtime. We propose use of a *multi-stage dynamic inference* mechanism, where two different bioformers, plus an optional non-deep classifier, are combined with the goal of reducing the average energy consumed by the system for a minimal accuracy drop. The details of this mechanism are provided in Section 4.4.

### 4.1. Target Dataset

To assess the performance of our proposed method, we employ the Ninapro DB6 [7], one of the most popular public datasets for sEMG-based hand gesture reconition, which was specifically designed to study the degradation over time of the classification accuracy due to the aforementioned non-idealities.

More in detail, the Ninapro DB6 includes data collected from 10 non-amputee subjects (7 males and 3 females with an average age of 27±6 years), each of whom was asked to repeat the same experiments over 10 different sessions. During each session, the subject was asked to repeat the same hand gesture 12 times, and sessions have been performed twice a day for 5 consecutive days, one in the morning and one in the afternoon.

The recorded gestures are seven different grasps that closely resemble daily life activities, plus a rest position. Each gesture repetition lasts around 6 s, followed by 2 s of rest. Concerning the hardware used for the data collection, the array of sensors employed is composed of 14 Delsys Trigno sEMG Wireless electrodes, placed on the higher half of the forearm to simulate an amputation of the lower half. Each sensor collects the data with a sampling rate of 2 kHz.

**Figure 2:**
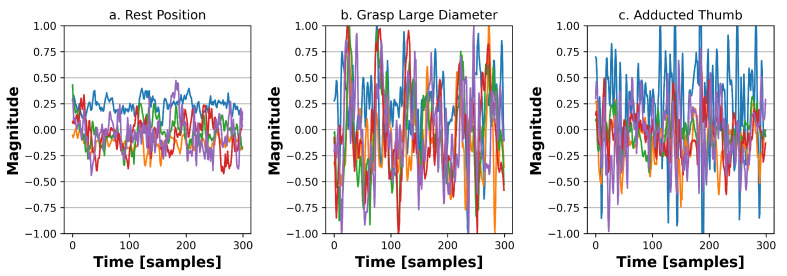
Examples of sEMG signals from [7] corresponding to different gesture classes.

In this work, we split the 10 recording sessions into two groups of 5 sessions each, the training dataset and the testing one. The training sessions always precede the testing ones to maintain temporal coherence among sessions (i.e., we used sessions 1–5 for training, and 6–10 for testing). We divided the data into windows of 150 ms (i.e., 300 samples) for preprocessing, with a time shift between them of 15 ms. The final input windows are of dimensions 300 (time samples) × 14 (sensors). Consistently with all previous works on this dataset, network training was performed on steady gestures, removing contraction transients, i.e., the first and last 1.5 s of each gesture. Examples of input windows fed to our neural networks are shown in Figure 2 for three different gestures.

### 4.2. Bioformer Architectures

**Figure 3:**
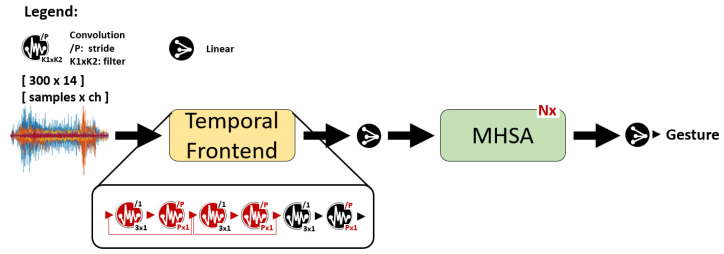
Architecture template of bioformers.

For the architectural template of bioformers, we take inspiration from ViTs, which have been shown to outperform CNNs on high-end computer vision tasks [22]. Similarly to ViTs, our architecture is structured as a chained connection of three main components: (i) an initial set of convolutional layers (*temporal frontend*), which extracts patches and local features from the input, (ii) one or more MHSA layers that combine patches to extract higher-level features, and finally (iii) a single fully-connected output layer, applied only to the first patch (a special *class token* prepended to the proper input sequence) to associate a gesture label to the input. A scheme is shown in Figure 3. As a key difference compared to ViTs, our frontend uses 1-dimensional convolutions, since we are dealing with sEMG time-series.

#### 4.2.1. MHSA Layer Details

**Figure 4:**
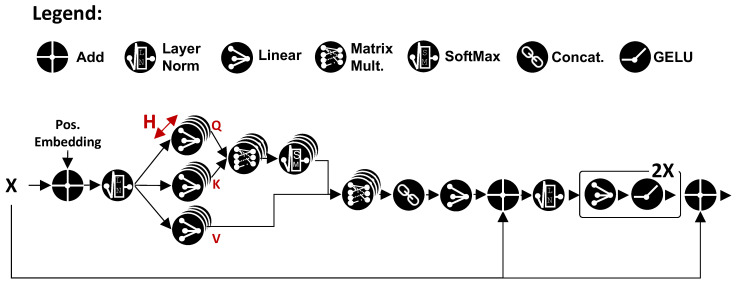
Multi-head self-attention layer implementation used in this work. All operations are applied to the entire sequence of input patches.

The scheme of the MHSA layer used in bioformers is shown in Figure 4. Given an input sequence of patches *X*, with dimensions *S* × *C*, with *S* being the sequence length and *C* the number of channels, the first operation is the addition of *positional embeddings*. These are vectors associated with each position in the sequence, that allow MHSA to take into account positional relations when extracting information from the patches. As in ViTs, we used positional embeddings *learned* during training.

After a layer normalization (LN), the next set of operations (shown as multiple overlapping circles) was repeated identically *H* times, with *H* being the number of MHSA *heads*. Namely, each head projects the input sequence into three different spaces of size $D_{H}$, yielding as output three vectors named *queries* (*Q*), *keys* (*K*), and *values* (*V*). The projections are computed as

(1) $$Q=XW_{query}\;K=XW_{key}\;V=XW_{value}$$

where $W_{query}$, $W_{key}$, and $W_{value}$ are matrices of size *C* × $D_{H}$. Subsequently, the computed vectors are fed to the *scaled dot-product* block, which is the proper attention layer, and combines queries, keys, and values with the following formula:

(2) $$Attention\left(Q,K,V\right)=SoftMax\left(\frac{QK^{T}}{\sqrt{D_{H}}}\right)V$$

The softmaxed matrix multiplied with *V* is the one containing attention weights, representing the relative importance of each element in *V* with respect to all the others.

Next, the concatenation of the attention outputs over the *H* heads is fed to a linear layer that re-projects them from the *S* × $\left(H\times D_{H}\right)$ space back to *S* × *C*. This permits the realization of a *residual connection*, which sums together the input and attention output for each sequence element. The residual sums undergo another LN, followed by two consecutive linear layers, each with a Gaussian error linear unit (GELU) non-linearity, where the first one increases the number of channels from *C* to $C^{\prime }>C$ and the second reduces it again to *C*. Lastly, another residual addition completes the block.

#### 4.2.2. Architecture Exploration

In order to explore the trade-off between task accuracy and complexity, we parametrized the bioformer template and explored it through a grid search. Specifically, the search is split into two phases, as shown on the left of Figure 1. At first, we explored the model architecture, making the depth of the convolutional frontend and of the attention component modular, and tuning some of the key hyper-parameters of the latter. During this phase, we considered bioformer variants that process “default” input patches of intermediate size. Then, in a second phase, we varied the patch size for the Pareto-optimal models obtained in phase 1, since this hyper-parameter greatly influences both accuracy and complexity. We separate the two phases to keep the cost of the search manageable.

We explore frontends composed of a minimum of 1 and a maximum of 6 1-dimensional convolutions (Conv). Each Conv is followed by batch normalization (BN) and by a rectified linear unit (ReLU) activation function, not shown in Figure 3 for simplicity. Specifically, we consider 1, 2, or 3 frontend blocks, with 1 or 2 layers each. The first (optional) Conv layer of each block has a 3 × 1 filter size, and a stride of 1. The second Conv uses a bigger stride, equal to the filter size, to process patches of the input signal [22,32]. The patch size is equal to the convolution stride, indicated as *P* in Figure 3. The three frontend blocks have 32, 16, and 14 output channels, respectively. To maintain the compatibility of tensor shapes with the case in which we have a single patching layer, the number of output channels of the last block is always 14, i.e., equal to the number of channels in the input sEMG signal. When using less than 3 blocks, we removed them from first to last (e.g., the 32-output-channels initial block is the first one to be removed). When a block contains 2 layers, the first convolution alters the number of channels, while the second one keeps them constant, while reducing the temporal dimension thanks to the stride. In the initial model architecture exploration phase, we consider patch dimensions ($P_{0}$, $P_{1}$, $P_{2}$) equal to ( , , 10), ( , 5, 3) and (1, 5, 3) for bioformers with 1, 2, and 3 frontend blocks, respectively. The larger patch size selected for 1-block frontends is motivated by the quadratic complexity of the attention block with respect to the input size sequence. We therefore selected patch sizes that ensure that the input sequence length passed to the MHSA part is always lower than 30 to keep a low complexity of the bioformer architecture.

For what concerns the attention part, we consider bioformers with either 1 or 2 MHSA blocks, structured as described in Section 4.2.1. We varied the number of attention heads *H* in the set {2, 4, 8}, and the size of each head’s projection ($D_{H}$) in {8, 16, 32}. The hidden dimensions of the two linear layers are fixed at C=64 and $C^{\prime }=128$. An additional linear layer before the first MHSA block projects the convolutional front-end output onto *C* channels.

Combining the MHSA and frontend variants, a total of 108 different bioformer models are considered in the first phase of the exploration. Note that although hyper-parameter ranges are determined by hand, we verified empirically that values outside these ranges lead to poorly performing or not converging models. In the results of Section 5, we report only a subset of the 108 models considered, i.e., those achieving interesting accuracy versus complexity trade-offs.

Once a set of Pareto-optimal bioformers with default patch sizes has been identified, we further explore the complexity vs. accuracy trade-off induced by varying *P* to obtain additional network configurations. In particular, we consider patch size values in {5, 10, 30, 60}, since values outside this range lead to worsening results for the specific models that end-up on the Pareto-frontier after the first exploration phase.

### 4.3. Training Protocol

We trained each model in the search space defined in Section 4.2 to completion, with a pre-training plus fine-tuning scheme similar to the one described in our previous work [23]. Namely, we initially pre-trained the model on the first 5 data collection sessions of *all* subjects for 100 epochs. We used the Adam optimizer and a standard categorical cross-entropy loss function. We set the batch size to 64, and applied a triangular cyclic learning rate schedule in the range $\left[10^{-7}:10^{-3}\right]$ with a period of 400 training steps.

We then fine-tuned the model on the data relative to the first 5 sessions relative to the target subject for 20 additional epochs. In this phase, we used a batch size of 8 and a fixed learning rate of $10^{-4}$, reduced by a factor of 10 after the first 10 epochs. All other training hyper-parameters are identical to those of the pre-training. The obtained model was finally tested on the last 5 sessions relative to the same subject, according to the reference per-patient split proposed in [7]. Fine-tuning and testing were repeated for each of the 10 subjects in the dataset.

We did not employ training techniques that require periodically evaluating the model on unseen data (e.g., early stopping, best-model checkpointing, etc.). Moreover, we considered the average accuracy over all patients when comparing different bioformer architectures, similarly to a k-fold cross-validation approach. Thus, we do not need a separate validation set, and we can include the entire first 5 sessions in the training sets for pre-training and fine-tuning. This is a key difference compared to our earlier work [23], and is the main reason why we sometimes achieve a higher test accuracy with an identical model architecture.

### 4.4. Dynamic Inference

The result of the architecture optimization and of the following Pareto analysis is a set of bioformers, each achieving a different test accuracy, and involving a different number of parameters and MAC operations per classification. In a standard machine learning operations (MLOps) flow, the model to deploy in-field would be chosen from this set depending on the specific requirements of the hardware (e.g., the most accurate one requiring less than a given amount of energy/latency). While this offers a certain degree of flexibility, designers still have to select among few options that provide a coarse-grain sampling of the design space, and most importantly, the choice is made *once-for-all*. Intuitively, this could be suboptimal.

In fact, one might want to run a complex yet accurate model only when it is (i) affordable (e.g., the system battery is well-charged) and (ii) necessary (the input to be classified is a “difficult” one—e.g., a particularly noisy sample, or one corresponding to a hard-to-recognize gesture, such as a grasp). Vice versa, when the battery is about to discharge, and/or when the input is “easy” (e.g., a sample corresponding to the rest class), one might prefer to use a simpler and less energy-hungry model.

This additional flexibility can be offered by *dynamic (or adaptive) inference* [2] techniques, which optimize the way ML applications operate *at runtime*, lifting the usual assumption that the same model must be executed identically for all processed inputs.

Many dynamic inference techniques have been proposed in recent years, mainly targeting CNNs [2,34,35,36,37,38,39,40,41,42,43]. One of the first yet most effective approaches is the so-called “big/little” system, in which two classifiers of different complexity and accuracy are combined at runtime to improve efficiency [34]. The basic idea is to use the “little” CNN for easy inputs, and the “big” one for difficult ones. However, since knowing the difficulty of an input a priori is difficult, an iterative approach is used instead: each new input is first classified by the “little” model, also computing a measure of prediction *confidence*. If the confidence score is high, the little model’s output is kept, and the prediction stops. Otherwise, the input is classified again using the “big” model. This introduces an overhead (two classifications for “difficult” samples). However, assuming that easy inputs are the majority, which is the case in most applications, the big/little system can outperform both individual models, achieving an accuracy similar to the “big” CNN with a lower *average* latency/energy per input, or vice versa, a higher accuracy than the “little” model, with a small latency/energy overhead. Refinements of this technique build the little model as a *portion* of the big one, e.g., deactivating part of its layers in the first iteration [35,36], reducing the data bit-width [37]. To our knowledge, the big/little scheme has not yet been applied to tiny transformers.

An alternative dynamic approach is *staged* inference, in which a task is partitioned in sub-problems solved sequentially by increasingly complex classifiers. The underlying principle is that not all classes are equally difficult to recognize [43]. Consequently, the initial stages only recognize inputs belonging to easy classes, outputting a “fallback class” for all other samples. Then, the following stages refine the classification for those inputs that were associated to the fallback class. As practical examples, in a diagnostic system, a patient can be classified as healthy or sick in stage 1, then refining the disease classification for sick patients in stage 2 [38]. In people counting, the first stage could perform human detection, and the second one could apply the counting algorithm only to frames that contain at least one person [40]. In a human activity recognition system for wearables, easy activities such as laying or sitting can be detected in stage 1, and more complex ones such as running or cycling in stage 2 [39].

We propose the application of *both* staged and big/little inference to sEMG-based gesture recognition. To our knowledge, the combination of these two approaches has not been studied in the previous literature. The resulting system is a 3-stage one, and combines two bioformers plus a shallow ML model. A block diagram is shown on the right of Figure 1.

#### 4.4.1. Rest Detector

The first stage is implemented with a small RF, and its goal is to classify an input into *rest* or *no-rest*. To train the RF, we used all inputs from [7] that do not belong to the *rest position* class as no-rest examples. The rationale for this first stage is two-fold: (i) rest samples would be the most frequent ones in a real deployment scenario and (ii) they correspond to sEMG signals with low variability, easily distinguishable from those associated with specific gestures. This can be seen by comparing the three charts of Figure 2, which shows three examples of sEMG signals corresponding to one rest input and two other gestures, taken from our target dataset (see Section 4.1).

We trained a different rest detector per subject, using the same data split described in Section 4.3. We considered RFs composed of at most 12 decision trees, each with a maximum depth of 5. We fed the model with a single feature per sEMG channel, corresponding to the total *waveform length* of the signal in an input window, i.e.,

(3) $$F_{i}=\sum_{k=1}^{300}∥X_{i,k}-X_{i,k-1}∥$$

where 300 is the number of samples in a window and $F_{i}$ is the resulting *i*-th channel feature. This feature is selected in accordance with other previous works that used classic ML models for this task [7,28,29], and also due to its simple extraction, which has linear complexity with respect to the window length. Since we have 14 sEMG electrodes (i.e., channels), the total number of features fed to the rest detector is 14. Note that the memory and number of operations required to run this model, including the features extraction, are negligible even compared to the simplest bioformer.

#### 4.4.2. Big/Little Bioformers

No-rest samples were then fed to the following stages, which consisted of a big/little classifier obtained combining two bioformers of different accuracy and complexity. The details of the selected architectures are provided in Section 5. The classification confidence metric that we used to decide between stopping after the “little” bioformer (stage 2) or continuing with the “big” one (stage 3) is the score margin (SM) [34], which can be computed with the following formula:

(4) $$SM={\hat{y}}_{i_{max}}^{\left(little\right)}-{\hat{y}}_{i_{2nd-max}}^{\left(little\right)}$$

where ${\hat{y}}_{i_{max}}^{\left(little\right)}$ and ${\hat{y}}_{i_{2nd-max}}^{\left(little\right)}$ denote, respectively, the largest and the second largest class probability scores produced by the little model. Then, at runtime, the SM is compared with a tunable threshold $T_{h}$ to perform the stopping decision:

(5) $$\hat{y}=\left\{\begin{array}{c} {\hat{y}}^{\left(little\right)}\mathrm{if}SM>T_{h}\\ {\hat{y}}^{\left(big\right)}\mathrm{otherwise} \end{array}\right.$$

The rationale of this stopping criterion, which is the most commonly used one in this kind of system, is that a large SM corresponds to a confident classification (only one class is highly probable), and vice versa.

Different criteria can be used to select the two bioformer models that form the big/little system. In general, the choice depends on the range of accuracy and energy/latency values that must be supported by the system at runtime. In absence of more specific constraints, a natural choice is to select the two extremes of the Pareto frontier in terms of accuracy versus number of operations, determined at the end of the architectural exploration phase. This is the approach followed in our experiments, as detailed in Section 5.3.

#### 4.4.3. Tuning Parameters and Overheads

The proposed dynamic system has several degrees of freedom that allow tuning of its configuration at runtime. The threshold $T_{h}$ of the big-little bioformers can be changed to easily achieve different trade-off points in terms of complexity and accuracy. For instance, a large $T_{h}$ stops the classification at stage 2 (“little” bioformer) only when the prediction is very skewed towards a single class, thus resulting in a *conservative* system, that prefers invoking the “big” model more often in order to maximize accuracy. Conversely, a small $T_{h}$ favors energy savings, stopping more frequently at the “little” stage. As shown in our results, varying $T_{h}$ permits many different runtime operating conditions to be obtained with just two models.

Another degree of freedom is offered by the possibility of *disabling* the RF-based rest detector. In fact, the two bioformers can still classify inputs as belonging to the “rest” class, hence they can operate alone. This creates another complexity/accuracy trade-off. In fact, while the filtering of “easy” inputs by the rest detector greatly reduces the average computation per input, it may also be detrimental for accuracy, since false positives (samples wrongly classified as “rest”) cannot be corrected by following stages. Thus, disabling the rest-detector moves the operating point towards higher latency/energy and higher accuracy regions of the space. Note that the fact that bioformers maintain the “rest” class also has the secondary benefit of possibly correcting some rest detector false negatives (samples wrongly classified as “no rest”).

Combining these two knobs (varying $T_{h}$ and disabling the RF) yields 10 s of different operating points, switchable at runtime, and spanning a wide range in terms of accuracy and energy/latency.

The evaluation of the early-stopping policy for the big/little bioformer has a negligible impact on the total prediction time and energy, and the memory required for storing the rest-detector is also extremely small. The main overhead of the dynamic system is the additional memory required for storing two bioformers instead of one. However, as will be discussed in Section 5, the resulting flexibility makes it acceptable. In fact, spanning a comparable range of operating conditions with multiple independent static models (not combined through a multi-stage dynamic approach) would involve significantly larger overheads.

## 5. Experimental Results

### 5.1. Setup

We trained and validated bioformer models on the NinaPro DB6 dataset (described in Section 4.1) using Python 3.8.13 and PyTorch 1.10.2. The initial training and architecture exploration was performed with a standard single-precision floating-point (fp32) representation for weights and intermediate activation tensors. Before deployment, we then performed quantization-aware training (QAT) to convert fp32 tensors into 8-bit integers (int8). Besides reducing the storage and memory required by Bioformers, this step is also necessary because our target edge platform does not include a hardware floating-point unit. We used the built-in QAT functionality of PyTorch for this step.

We deployed the quantized models on the GAP8 SoC [15] using the optimized library of kernels (i.e., layers implementations) described in [44] for MHSA, and in [45] for convolutions. GAP8 is a commercial SoC from GreenWaves Technologies, which inlcudes a controller unit called fabric controller (FC), composed of a single RISC-V core, which manages the peripherals and orchestrates the program execution, and a cluster of 8 identical RISC-V cores (with a shared 64 kB scratchpad memory) which accelerates intensive workloads. FC and cluster share a 512 kB L2 memory. We refer to [15] for more details on the platform.

We compared our bioformers against the CNN-based gesture recognition solution proposed in [24], called TEMPONet, which achieved the previous state-of-the-art accuracy on NinaPro DB6. To make the comparison fair, we used an identical training protocol for our models and for TEMPONet, including the pre-training plus fine-tuning scheme which was not used in the original work of [24].

### 5.2. Architecture Exploration

**Figure 5:**
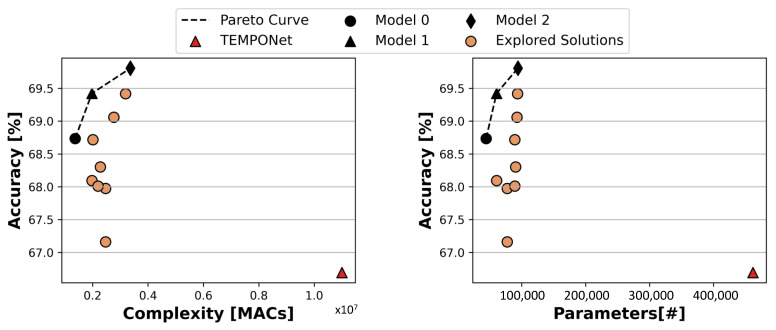
Network topology exploration results.

Figure 5 shows the results of the first part of the bioformer architecture exploration. As explained in Section 4.2, we first explored bioformer variants using default patch sizes, varying hyper-parameters such as the number of layers in the convolutional frontend, the number of MHSA blocks, the number of attention heads, and the size of each head’s projection.

The two graphs of Figure 5 show the Pareto curves generated by some of these architectures in terms of accuracy versus number of MAC operations and accuracy versus the number of parameters, respectively. Accuracy results refer to floating-point models at this stage. Each marker represents a specific architecture configuration. Note that only the configurations that are close to the Pareto frontier are shown in the Figure, while models with low accuracy (or not converging), too high complexity, or too large size are not reported to ease the visualization.

Pareto-optimal solutions are highlighted in the two graphs by black dashed lines. Interestingly, the same set of three models turns out to be optimal when considering both MACs and parameters as complexity metrics. We name these three bioformers, respectively, Model 0 (identified with marker • in the graphs), Model 1 (▴), and Model 2 (⧫), in order of increasing accuracy. With default patch sizes, these three models span a relatively small accuracy range in 68.73–69.81%, while MAC operations vary more widely in 1.37–3.36 M, and parameters in 44.35–94.09 k. The detailed architecture configurations of the three Pareto-optimal bioformers are as follows:

- Model 0: H=8, $D_{H}=8$, 1 block with 1 Conv layer in the frontend;
- Model 1: H=8, $D_{H}=16$, 1 block with 1 Conv layer in the frontend;
- Model 2: H=8, $D_{H}=32$, 1 block with 2 Conv layers in the frontend;

Several interesting insights can be drawn from these results. First, a large number of heads *H* in MHSA blocks is important to achieve high accuracy on this task. Second, increasing the size of each projection ($D_{H}$) is the easiest way to trade-off more operations and parameters for more accuracy. Last, a deep temporal frontend is not necessary, since all Pareto-optimal solutions use a single frontend block, and at most two one-dimensional convolutional layers.

All three bioformers significantly outperform the comparison baseline (TEMPONet) [24], shown as a red triangle in the Figure. Importantly, the advantage of bioformers comes mostly from their ability to learn effectively from larger amounts of training data. In fact, as shown in our previous work of [23], the pre-training plus fine-tuning scheme yields greater benefits on transformer-based architectures compared to TEMPONet. More in detail, in [23], TEMPONet with pre-training still outpeformed our best bioformer in terms of accuracy, although at the cost of many more parameters and operations. In this paper, however, thanks to the wider architecture exploration, and to the slightly different training protocol, which uses *all* data from the first five sessions of a subject as a training set, we obtain transformers that are both more accurate and less computationally demanding compared to TEMPONet. Also note that the highest accuracy of 69.81% that we were able to attain on the DB6 dataset represents the most recent state-of-the-art for trainings that use a data split based on time (i.e., using only data from the past to infer gestures at future time instants). While there are still ample margins for improvement, this accuracy is sufficient for various practical tasks. For example, a “majority voter” on the most recent N predictions might be utilized to practically achieve much higher accuracy while only slightly increasing the prediction latency in a system for controlling a robotic arm. The errors, in fact, tend to be random and evenly distributed, as analyzed in earlier publications [24]. In other words, with a 69.8% accuracy, an identical gesture is expected to be misclassified three times, evenly distributed in between seven accurate classifications rather, than for instance, ten consecutive times. In this situation, a majority voter could completely weed out mistakes.

**Figure 6:**
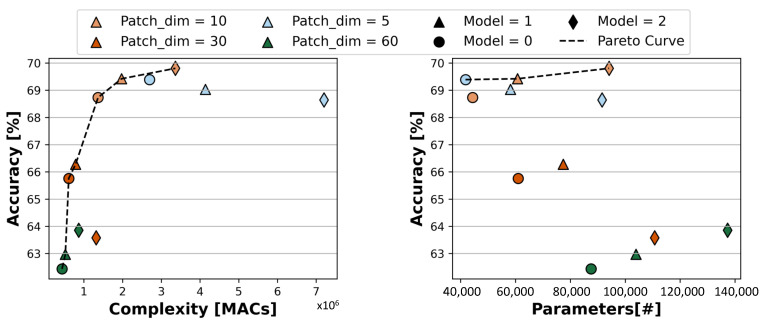
Patch size exploration results.

Figure 6 shows the results obtained exploring the patch size hyper-parameter. Namely, the results are obtained starting from the three Pareto-optimal bioformers with default patch size shown in Figure 5, and varying the patch size generated by the single frontend block in the set {5,10,30,60} (the default value was P=10). The graph axes are analogous to those of Figure 5. Each Pareto model is represented by a different marker shape, while different colors denote different patch sizes. The Pareto frontiers are again highlighted by black dashed lines.

Varying the patch size significantly enriches and enlarges the set of optimal solutions in the accuracy versus MACs space, which now includes models with an accuracy ranging in 62.43–69.81% and requiring 0.44–3.36 M MAC operations. In contrast, the effect is less evident when considering the accuracy versus parameters space, where Pareto-optimal models have accuracies very close to each other (69.39–69.81%) and include 41.8–94.09 k parameters.

This is expected, since the number of operations in the forward pass of an MHSA block is quadratic with respect to the sequence length *S*, which is a direct function of the patch size *P* (larger patches result in a shorter sequence and vice versa). The operations in the temporal frontend are independent from *P*, since the (optional) first convolution has a fixed 3 × 1 filter, and the second one performs a single MAC per input value, regardless of *P* (due to using a stride equal to the kernel size). Overall, we therefore obtain an inverse dependency, where models with larger patch sizes require fewer operations, and generally achieve lower accuracy.

Instead, the number of weights in MHSA blocks is not affected by *P*, given that *C*, $C^{\prime }$, *H*, and $D_{H}$ are fixed. The only place where the patch size affects the parameters is the strided convolution in the frontend. In fact, the number of parameters of this layer is expressed by the formula $C_{in}\;\cdot \;C_{out}\;\cdot \;K1\;\cdot \;K2=C_{in}\;\cdot \;C_{out}\;\cdot \;P\;\cdot \;1$, which is linearly dependent on *P*. So, in this case, a larger patch size corresponds to a larger layer, but the impact on the total size of the model is minimal. Nonetheless, we still find a model that improves the previous Pareto frontier (corresponding to the orange markers). Namely, for Model 0, a patch size of 5 slightly improves accuracy, due to better generalization, compared to the case of P=10, while requiring fewer parameters.

In summary, this exploration shows that all three bioformers (Model 0, 1 and 2) remain in the Pareto front when varying *P*. Further, increasing the patch size is a very effective way to trade-off a reduction in the number of inference operations for a drop in accuracy. When the goal is to reduce the memory occupation (i.e., the number of parameters), instead, the patch size should be reduced as much as possible.

### 5.3. Dynamic Inference

**Figure 7:**
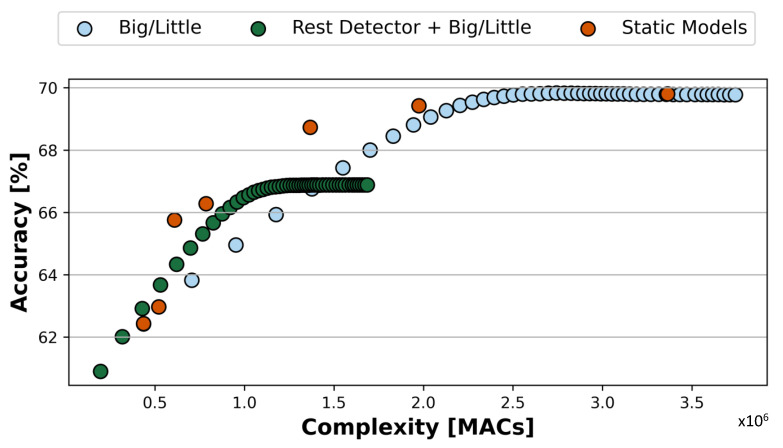
Dynamic inference results.

Figure 7 shows the results obtained by the dynamic inference system described in Section 4.4. In particular, the system is built selecting the two bioformers with the least/most MAC operations as a basis for the big/little scheme. Those correspond to Model 0 with P=60 and Model 2 with P=10.

The green and blue points in Figure 7 correspond to the possible operating conditions of the dynamic inference systems with/without the RF rest detector enabled, respectively. Different points are obtained varying in [0:1] the confidence threshold $T_{h}$ of the big/little scheme, which determines when to stop after stage 2, or continue with stage 3. For comparison, Figure 7 also reports the results of independent Pareto-optimal static models spanning a comparable range of MACs, directly derived from Figure 6, as orange dots. The number of MAC operations reported for the dynamic approach is the *average* over the test set for all subjects.

As shown in the Figure, varying $T_{h}$ allows us to easily obtain many different operating points in the accuracy versus number of operations plane. Specifically, large $T_{h}$ values yield high accuracy at the cost of more operations and vice versa. In addition to that, enabling or disabling the rest detector creates two different sets of operating points, one more efficient and less accurate (with rest detector enabled, shown in green) and the other more accurate but requiring a larger number of operations per input on average (rest detector disabled, in blue).

Overall, combining these two parameters, the three-stage dynamic system can span a wide range in accuracy 60.90–69.78% and MACs (196.4 k–3.74 M), which then translate into different average energy consumption and latency values. In total, the two combined knobs result in ≈100 distinct operating points. Compared to static solutions, the dynamic achieves slightly inferior but comparable accuracy versus MAC trade-offs. However, the advantage of a dynamic approach with respect to deploying all static models and selecting among them at runtime depending on external constraints (e.g., battery status) becomes evident when considering the memory overhead, as discussed below in Section 5.4. Intuitively, the dynamic system can span this wide trade-off with only two bioformers (plus the negligible rest detector). In contrast, to reach a similar range of accuracy and complexity based on the standalone static models (orange dots) would require deploying seven different bioformers with obvious overheads in terms of memory and storage. Yet, the flexibility offered by the latter approach would still be more limited (only 7 points versus 100).

### 5.4. Deployment on GAP8

We deployed all optimal bioformer architectures in terms of accuracy versus MACs derived in Figure 6 on the GAP8 SoC, after quantizing them to 8-bit. For comparison, we also quantized and deployed the TEMPONet CNN from [24]. Table 2 shows the detailed deployment results. Besides the gesture recognition accuracy, we report the memory occupation, energy consumption, and inference latency of each model on the target hardware (HW). Moreover, we also report the total MACs per inference in millions (MMAC), and two measures of HW efficiency, i.e., the GMAC/s and the GMAC/s/W. Note that accuracy values in the table are lower than those in previous figures due to the effect of quantization.

All bioformers with default patch size (P=10) outperform TEMPONet in terms of accuracy (from +3.3% to +5.1%), while at the same time significantly reducing energy consumption and inference latency (from 7.8× to 19.2×). When enlarging the patch size, the energy savings increase. For instance, with respect to [24], Model-0 bioformers with P=30 and P=60 reduce the energy consumption by 44.5× and 58.6×, at +2% and −1.1% accuracy, respectively. Notably, after quantization, the accuracy ranking of the three bioformers with default patch sizes reverses. However, since the accuracies of these three models are very close to each other, this can be attributed to the random noise inserted by quantization.

The fact that the total number of MMAC is 4.8× smaller than those required by TEMPONet, even for the largest bioformer, hints that similar energy and latency reductions could be achieved regardless of the target HW platform. However, the GMAC/s and GMAC/s/W columns show that, in the case of GAP8, our models not only require fewer total operations but also utilize the hardware better, achieving higher throughput and efficiency. Concerning real-time constraints, all bioformers can provide very fast responses with a latency of <3 ms, well below the 15 ms constraint imposed by the time lag between two consecutive input windows in Ninapro DB6.

Lastly, bioformers are also small. Model-0 with P=10 only requires 44.35 kB, thus being 10.4× smaller than TEMPONet, and simultaneously more accurate (+5.1%). All our models easily fit in the 512 kB L2 memory of the target SoC (occupying at most 20% of it), whereas the baseline CNN fills it almost entirely.

Table 3 reports the results obtained deploying several dynamic inference solutions on GAP-8. Configurations are described according to the model used for each stage (leftmost three columns of the table). For bioformers, we use the notation-N-(P), where N is the model number (0, 1, or 2) and P the patch size. Solutions that do not use all the stages have “n.a.” values in some of the columns. The “Technique” column clarifies the type of dynamic system considered, where “Rest” corresponds to the usage of the RF-based rest detector, and “Big/Little” to the combination of two different bioformers. We report the accuracy, MMAC, latency, and energy consumption for each configuration, where the last two metrics are averaged over the Ninapro DB6 test sets.

The first three rows report the metrics relative to the three static models that we used as the basis for big/little bioformers, for comparison. Then, the following six rows report the metrics obtained by two dynamic systems constructed using these bioformers as building blocks. Precisely, we use the least-energy-consuming bioformer (Model-0 with P=60) as the “little” neural network (NN) in all cases, and we consider as a “big” NN either the bioformer that achieves the highest float accuracy (Model-2 with P=10) or the one achieving the highest int8 accuracy (Model-0 with P=10). The former results in the same combination of big/little models shown in Figure 7. Each of the two dynamic systems is shown in multiple configurations, namely, (i) a solution that combines the rest detector with the big model directly, without leveraging the big/little technique; (ii) the big/little system with rest detector disabled; (iii) the big/little system with rest detector enabled. Since each of these combinations can work in tens of different operating conditions (obtained bchanging $T_{h}$), we report the latency and energy results corresponding to the $T_{h}$ value that yields the maximum accuracy with the fewest MACs, i.e., the equivalent of the leftmost extreme of the horizontal green and blue segments in Figure 7.

The results show that, with almost the same accuracy of the big models from which they are generated (67.02% vs. 67.04% and 65.20% vs. 65.28%, respectively, for Model-0 and Model-2 as “big” NNs), big/little bioformers can reduce the average inference energy consumption by 1.03×–1.35× (56 μJ vs. 58 μJ and 105 μJ vs. 143 μJ, respectively). Furthermore, the effectiveness of the proposed three-stage solution is demonstrated by the fact that it achieves the same accuracy as a system that only uses the RF rest detector combined with the “big” NN (64.72% vs. 64.69% and 62.54% vs. 62.55% for the two cases) while reducing the average consumption per input by 1.07×–1.31×. Therefore, we can conclude that our multi-stage dynamic approach combining staged and big/little inference can further reduce energy consumption and enhance flexibility compared to any scheme involving only two levels. Furthermore, note that these results are obtained with the NinaPro DB6 test set, which includes a comparable number of “rest” and “grasp” samples. In a real deployment scenario, the amount of “rest” samples would probably be much larger, thus resulting in larger energy savings for the dynamic system.

In terms of memory occupation, the entire three-stage system requires a total of 132 kB/ 184 kB when Model-0 and Model-2 are used as “big” NNs, respectively. While the memory overhead compared to a single static model is not negligible, the system still largely fits the L2 memory of GAP8. Conversely, for instance, the total memory occupied by all the seven optimal static solutions reported in Table 2 is 529.04 kB, i.e., 2.90× more than our largest dynamic system, and exceeding the L2 of GAP8. Thus, besides allowing a finer-grain tuning of the operating point, the dynamic method is also much less demanding to deploy compared to multiple independent models.

## 6. Conclusions

We have proposed bioformers, a set of efficient tiny transformers for sEMG-based gesture recognition at the edge, that achieve state-of-the-art accuracy on the popular Ninapro DB6. With an extensive architecture exploration, we obtained a rich set of trade-offs in terms of accuracy versus complexity. When deployed on the GAP8 edge SoC, bioformers outperform a previous CNN-based solution [24] in accuracy, while also occupying less memory, and requiring lower latency and energy.

Furthermore, we have proposed a novel multi-stage dynamic inference solution that combines staged and big/little inference, in order to further improve energy efficiency. Thanks to dynamic inference, our system can span a wider range of trade-offs in terms of accuracy and computational complexity, only by varying two run-time configurable parameters, and achieves an additional energy saving on GAP8 with respect to static bioformers for the same accuracy. Overall, our results show that the proposed gesture recognition system based on bioformers and dynamic inference enables continuous on-device execution in real time, with very low energy consumption.

## Figures and Tables

**Table 1:** Previous works tested on Ninapro DB6 dataset [7]. Accuracy results preceded by “q” refer to models using a int8-quantized data representation.

Work	Year	Features	Algorithm	Real-Time	Emb.	Accuracy Inter % / Intra % / Random %	Energy
Palermo [7]	2017	WL	RF	No	No	25.4 / 52.4 / n.a.	n.a.
Cene [33]	2019	MAV, VAR, RMS	ELM	No	No	41.8 / 69.8 / n.a.	n.a.
Zanghieri [24]	2019	Raw Data	TCN	Yes	Yes	65.2 (q: 61.0) / 71.8 / n.a.	0.90 mJ
Wei [25]	2019	Raw Data	Multi-View CNN	No	No	64.1 / n.a. / n.a.	n.a.
Zou [26]	2021	Raw Data	Multiscale CNN	No	No	n.a. / n.a. / (97.2, 74.5, 90.3) *	n.a.
Han [27]	2021	Raw Data	Multiscale CNN	No	No	n.a. / n.a. / 98.52 *	n.a.
Bioformer [23]	2022	Raw Data	Transformers	Yes	Yes	65.7 (q: 64.7) / n.a. / n.a.	0.139 mJ
**Our Work**	**2023**	**Raw Data**	**Transformers**	**Yes**	**Yes**	**69.8 (q: 67.0)** / **n.a.** / **n.a.**	**0.143 mJ**

* Training and testing data are randomly divided without following temporal order.

**Table 2:** Performance of Pareto-optimal bioformers on GAP8.

Model	Patch Size	Memory	MMAC [#]	Lat. [ms]	Energy [mJ]	GMAC/s	GMAC/s/W	Accuracy
- 0 -	10	44.35 kB	1.37	1.130	0.058	1.21	23.78	67.04%
- 1 -	10	60.74 kB	1.97	1.611	0.082	1.22	23.97	66.89%
- 2 -	10	94.09 kB	3.36	2.797	0.143	1.20	23.58	65.28%
- 0 -	30	60.99 kB	0.61	0.490	0.025	1.25	24.41	63.91%
- 1 -	30	77.38 kB	0.79	0.633	0.032	1.24	24.34	64.37%
- 0 -	60	87.55 kB	0.44	0.364	0.019	1.20	23.43	60.82%
- 1 -	60	103.94 kB	0.52	0.438	0.022	1.19	23.27	61.25%
[24]	n.a.	461 kB	16.00	21.828	1.113	0.73	14.37	61.95%

**Table 3:** Performance of dynamic inference solutions on GAP8.

Stage-1	Stage-2	Stage-3	Technique	MMAC [#]	Latency [ms]	Energy [mJ]	Accuracy
n.a.	n.a.	- 0 - (10)	None	1.37	1.130	0.058	67.04%
n.a.	n.a.	- 2 - (10)	None	3.36	2.797	0.143	65.28%
n.a.	n.a.	- 0 - (60)	None	0.44	0.364	0.019	60.82%
RF	- 2 - (10)	n.a.	Rest.	1.686	1.402	0.071	62.55%
n.a.	- 0 - (60)	- 2 - (10)	Big/Little	2.483	2.067	0.105	65.20%
RF	- 0 - (60)	- 2 - (10)	Rest. + Big/Litte	1.274	1.061	0.054	62.54%
RF	- 0 - (10)	n.a.	Rest.	0.685	0.570	0.029	64.69%
n.a.	- 0 - (60)	- 0 - (10)	Big/Little	1.319	1.098	0.056	67.02%
RF	- 0 - (60)	- 0 - (10)	Rest. + Big/Litte	0.631	0.525	0.027	64.72%

## Data Availability

No new data were created or analyzed in this study. Data sharing is not applicable to this article.
